# An experimental study on the use of BERS-2 test to diagnose behavior and emotion: (a model for teenagers)

**DOI:** 10.1192/j.eurpsy.2025.1099

**Published:** 2025-08-26

**Authors:** K.-U. Mijgee, Y. Amanbyek

**Affiliations:** 1 Psychological Service Center, Mongolian National University of Education, Ulaanbaatar, Mongolia

## Abstract

**Introduction:**

This study focuses on studying the most common psychological and physiological phenomena of adolescents, which directly and indirectly affect adolescent behavior and emotions, especially school children, using the BESR-2 test. Youth and peer pressure, crime, family and school stress, drug use, alcohol use, smoking, rape, theft, etc., create anxiety and psychological causes for teenagers in our society. In order to prevent and reduce the negative impact on the behavior and emotional state of the youth, it is imperative to study the current problems and find better solutions. In relation to this social need, the purpose of this study is to conduct a pilot study on how to diagnose the behavior and emotional state of adolescents using the BERS-2 test.

**Objectives:**

When analyzing the relevant theories and principles of the BERS-2 test, the research selects appropriate methods and methodologies (1) review and analyze the theoretical basis of the author’s use of the test; (2) examine the internal reliability and convergent validity of the BERS-2; (3) use and examine internal reliability and convergent validity; and finally (4) identify research limitations and future research directions.

**Methods:**

1,812 students from Ulaanbaatar and the local area, 1,812 teachers, and 1,812 parents, a total of 5,436 participants are participating in the pilot study. In this study, one of the most widely used strengths assessment tools in social services is the Behavioral and Emotional Rating Scale-2 (BERS-2), which includes separate rating scales for youth, parents, and teachers. Although the three assessment forms are similar, a few items have been slightly worded to better reflect the views of Mongolian youth, parents, and teachers. The Interpersonal Strengths subscale (14 items) measures a child’s ability to interact with others in social situations. Family involvement (10 items) assesses the child’s relationship with the family. The internal self-efficacy subscale (11 items) focuses on how the child perceives his or her own functioning. The school functioning subscale (9 items) assesses the child’s performance and abilities in school. The Power Influence subscale (7 items) measures a child’s ability to exert and receive influence from others.

**Results:**

The main results of the study are presented as follows.

1. Cronbach’s alpha coefficient for test validity was 0.816.
2. Behavioral and emotional indicators of the surveyed teenagers are 34.56% on average.
3. In terms of self-esteem of teenagers, their behavioral and emotional indicators are 47.6% or average; parents - 33.3%; teachers are 29.6%.

**Conclusions:**

it was observed that the superiority of emotions and attitudes is weak for middle class students. In other words, Mongolian children believe that it is necessary to develop emotional and behavioral strengths.

**Disclosure of Interest:**

None Declared

